# Zoonotic importance of canine scabies and dermatophytosis in relation to knowledge level of dog owners

**DOI:** 10.14202/vetworld.2015.763-767

**Published:** 2015-06-21

**Authors:** Heli S. Raval, J. B. Nayak, B. M. Patel, C. M. Bhadesiya

**Affiliations:** 1 Department of Veterinary and Animal Husbandry Extension, College of Veterinary Science and Animal Husbandry, Anand Agricultural University, Anand, Gujarat, India; 2 Department of Veterinary Public Health, College of Veterinary Science and Animal Husbandry, Anand Agricultural University, Anand, Gujarat, India; 3 Department of Extension Education, B.A. College of Agriculture, Anand Agricultural University, Anand, Gujarat, India; 4 Department of Veterinary Medicine, College of Veterinary Science and Animal Husbandry, Anand Agricultural University, Anand, Gujarat, India

**Keywords:** canine scabies, dermatophytosis, dog owners, Gujarat, knowledge level, zoonotic importance

## Abstract

**Aim:**

The present study was undertaken to understand the zoonotic importance of canine scabies and dermatophytosis with special reference to the knowledge level of dog owners in urban areas of Gujarat.

**Materials and Methods:**

The study was carried out in randomly selected 120 dog owners of 3 urban cities (*viz*., Ahmedabad, Anand and Vadodara) of Gujarat state, India. Dog owners (i.e., respondents) were subjected to a detailed interview regarding the zoonotic importance of canine scabies and dermatophytosis in dogs. Ex-post-facto research design was selected because of the independent variables of the selected respondent population for the study. The crucial method used in collecting data was a field survey to generate null hypothesis (Ho_1_). Available data was subjected to statistical analysis.

**Results:**

The three independent variables, *viz*., extension contact (r=0.522**), mass-media exposure (r=0.205*) and management orientation (r=0.264**) had significant relationship with knowledge of dog owners about zoonotic diseases. Other independent variables, *viz*., education, experience in dog keeping and housing space were observed to have negative and non-significant relationship with knowledge of dog owners about zoonotic diseases.

**Conclusion:**

Extension contact, exposure to extension mass-media, management orientation and innovation proneness among dog owners of 3 urban cities of Gujarat state had significant relationship with knowledge of dog owners on zoonotic aspects of canine scabies and dermatophytosis. Data provided new insights on the present status of zoonotic disease-awareness, which would be an aid to plan preventive measures.

## Introduction

Zoonotic diseases are those which can be passed from animals to humans and vice-versa. People frequently get exposed to the bacteria, fungi, viruses, and parasites of animal species dwelling around human population that cause zoonoses in a number of ways. Hence, it becomes important for people working with or handling animal to know about potential zoonotic disease transmission and preventive measures against them.

Little consideration has been given to the known and potential zoonotic infectious diseases of small companion animals. Dogs closely share the domestic environment with human and act as reservoir or source of many zoonotic diseases [1]. Humans usually serve as accidental hosts that acquire the disease through close contact with an infected animal, who may or may not be symptomatic. Children are at highest risk for infection because they are more likely to have close contact with pets. The route of transmission can be through the feces, urine, saliva, nasal discharge of the dog. Although dogs have been implicated in the transmission of zoonoses to their owners, the risk of transmission from contact with dogs is low and may be further reduced by simple precautions [2,3].

Studies on the importance of zoonotic infections with special reference to the knowledge level of dog owners in urban areas are lacking in Gujarat. Among others, canine scabies and dermatophytosis are two major dermatological afflictions in dogs, which cause zoonotic infection in people involved in dog-ownership as well as in professionals involved in pet practice [4]. The major factor contributing to the appearance of these zoonotic diseases in human population is increased contact with their pet animals. Limited large-scale studies have been made in India for assessment of the knowledge level of dog owners to prevent zoonotic infections [5].

The present study was undertaken to understand the zoonotic importance of canine scabies and dermatophytosis with special reference to the knowledge level of dog owners in Ahmedabad, Anand and Vadodara districts of Gujarat state.

## Materials and Methods

### Ethical approval

Present survey work specifically dealt with the knowledge level of dog-owners of urban areas regarding the zoonotic importance of canine scabies and dermatophytosis. The study did not include clinical trials on animals. The authors have taken informed consent of the participants.

### Study area

Giving due considerations to the numbers and an appropriate direct approach to interview dog owners, the present investigation was carried out in Ahmadabad (Geographic coordinates: 23.03°N 72.58°E), Anand (Geographic coordinates: 22.556°N 72.951°E) and Vadodara district (Geographic coordinates: 22.18°N 73.12°E) of Gujarat state (India) under the jurisdiction of the Anand Agricultural University, Anand.

For a selection of the respondent, a list of the dog owners was obtained from Government Veterinary Polyclinics of three urban areas where respondent visited or came for treatment of their dogs during last 3 years. Based on information from available resources, 40 dog owners were randomly selected from each town. Thus, a total of 120 dog owners were selected as respondents for the study and were subjected to an interview concerned with the knowledge level of dog owners to prevent zoonotic canine scabies and dermatophytosis. Ex-post-facto research design was selected because of the independent variables of the selected respondent population for the study. The crucial method used in collecting data was a field survey. The interview schedule was used as a tool for collection of requisite information in terms of percentage analysis. To know the relationship null hypothesis (Ho_1_) was also formulated.

### Statistical analysis

The data obtained were subjected to statistical analysis as described by Snedecor and Cochran [6], which included calculation and interpretation based on standard deviation and correlation coefficient (‘r’) value. Variables with p<0.05 were considered as statistically “significant”, variables with p<0.01 were considered as statistically “highly significant” while variables with p>0.05 were considered as statistically “non-significant”.

## Results and Discussion

Distribution of dog owners according to their knowledge regarding canine scabies is shown in Table-1. It was observed that more than half of the respondents possessed lower knowledge level about the disease. Out of 120, 100 (83.33%) were aware only of reddish discoloration of the skin in clinical cases of scabies. Furthermore, 88 (73.33%) and 73 (60.83%) respondents were aware about sites of clinical lesions of canine scabies in man and dogs, respectively. Only 67 (55.83%) respondents were aware about zoonotic transmission of canine scabies through direct contact. Canine scabies is a contagious disease caused by mite *Sarcoptes scabies* in dog and human. Since strains of *Sarcoptes scabies* are strictly host specific, human scabies of animal origin is usually superficial and self-limiting. The clinical and epidemiological features of canine scabies in human are history of contact with infested animal, sudden appearance of lesion, papulo-vesicular eruption with intense pruritus/itching, and absence of burrows with lesion only on exposed parts of the body.

**Table-1:** Distribution of the dog owners according to their knowledge about scabies (n=120).

Knowledge about scabies on	Number of respondents	Percentage
Scabies has lesions on dogs such as papule, vesicle on head, neck, ear flaps, face, brisket, snout, elbow, thigh, root of tail, etc.	73	60.83
Scabies has lesions on man such has vesicle on hand, finger, forearm, chest, thigh, leg, navel, penis and inguinal area	88	73.33
Mites (*Sarcoptes scabies*) on the body of dogs can be transmitted through direct contact or with contaminated fomites	67	55.83
Dogs skin becomes reddish	100	83.33
If animal rub on wall or carpet man get infection	79	65.83

Distribution of dog owners according to their knowledge regarding dermatophytosis is shown in Table-2. Out of 120, 98 (81.66%) possessed knowledge about types of skin lesions in dogs with dermatophytosis. Furthermore, 73 (60.83%) dog owners were aware about transmission of dermatophytosis from dogs to humans by direct contact. A lower population of dog owners (n=20, 16.66%) was reported to possess knowledge about the role of fomites in the transmission of dermatophytosis. Dermatophytosis is the disease of human and animals caused by fungi belonging to a group referred as dermatophytes. The disease is commonly known as ringworm and tinea. It has immense zoonotic importance as the agent cause ringworm in almost all animal species and human. Direct contact with the infected host is the most common mode of the transmission of the infection. Inanimate objects carrying fungal spores may also lead to infection. It is estimated that about 80% of the cases of the human ringworm in rural areas and about 10% urban areas are of animal origin. In dogs *Microsporum canis* infection may spread to human. Children are particularly susceptible and are often affected due to their frequent contact with dogs.

**Table-2:** Distribution of the dog owners according to their knowledge about dermatophytosis (n=120).

Knowledge about dermatophytosis on	Number of respondents	Percentage
Dermatophytosis/ring worm infection causes circular, diffuse, extensive lesions on skin of head, neck, face of dogs	98	81.66
Dermatophytosis/ring worm infection can be transmitted to dog owners through close contact with infected dogs	73	60.83
Dermatophytosis/ring worm infection can be transmitted to dog owners through contaminated fomites	20	16.66

Percentage analysis of profiles of dog owners is shown in Table-3 while statistical interpretation is shown in Table-4. Relationship between the profile of the dog owners and their level of knowledge about zoonotic diseases is discussed hereunder.

**Table-3:** Profiles of the dog owners.

Type of variable	Number	Percent
Personal variable		
Educational level		
Illiterate	1	0.85
Primary level (up to 7^th^ standard)	10	08.33
Secondary level (8-10^th^ standard)	18	15.00
Higher secondary level (11-12^th^ standard)	26	21.66
Graduate level	45	37.50
Post graduate level	20	16.66
Total	120	100.00
Experience in dog keeping		
Very low (up to 5 years)	37	30.83
Low (6-10 years)	55	45.83
Medium (11-15 years)	15	12.50
High (16-20 years)	8	06.67
Very high (above 20 years)	5	04.17
Total	120	100.00
Socio-economic variable		
Housing space		
Marginal housing space (up to1500 sq. ft.)	28	23.33
Small housing space (1501-2000 sq. ft.)	52	43.84
Medium housing space (2001-2500 sq. ft.)	25	20.83
Large housing space (more than 2501 sq ft.)	15	12.50
Total	120	100.00
Communicational variables		
Extension contact		
Very low (up to 7 score)	48	39.87
Low (8-14 score)	34	28.50
Medium (15-21 score)	10	08.30
High (22-28 score)	22	18.35
Very high (29-35 score)	6	04.98
Total	120	100.00
Mass-media exposure		
Very low (up to 4 score)	25	20.83
Low (5 and 8 score)	40	33.33
Medium (9 and 12 score)	30	25.00
High (13 and 16 score)	15	12.50
Very high (above 16 score)	10	08.34
Total	120	100.00
Psychological variables		
Management orientation		
Very low (up to 13 score)	0	0.00
Low (14-26 score)	10	08.33
Medium (27-39 score)	25	20.83
High (40-52 score)	65	54.17
Very high (53-65 score)	20	16.67
Total	120	100.00

**Table-4:** Relationship between profile of the dog owners and their level of knowledge about zoonotic diseases (n=120).

Profile of dog owners	Correlation coefficient (r value)
Education	−0.120 NS
Experience in dog keeping	−0.069 NS
Housing space	−0.165 NS
Extension contact	0.522**
Mass media exposure	0.205*
Management orientation	0.264**

NS=Non-significant,

* significant at p<0.05,

** significant at p<0.01

### Education and knowledge

The level of education of dog owners had negative and non-significant (NS) relationship (r=−0.120 NS) with their knowledge of zoonotic diseases (Table-4). The result signifies that level of education did not play any role in increasing or decreasing knowledge of the owners. It means that level of knowledge about zoonotic diseases was found identical amongst the dog owners with their respective level of education these findings are in correlation with Bhadesiya and Raval [5]. Thus, null hypothesis (H0_1_) in case of education was accepted and concluded that level of education of dog owners was the trivial factor for their knowledge of zoonotic diseases.

### Experience in dog keeping and knowledge

The experience in dog keeping of owners had negative and non-significant relationship (r=−0.069 NS) with their level of knowledge of zoonotic diseases (Table-4). The result showed that level of knowledge about zoonotic diseases was observed similar among all groups in relation to varied experience level. Thus, null hypothesis (Ho_1_) was accepted in case of experience in dog keeping and thus, it was concluded that there was non-significant influence of experience of dog owners in establishment of knowledge about zoonotic diseases [7-9].

### Housing space and knowledge

The housing space of the dog owners establishes negative and non-significant relationship (r=−0.165 NS) with their knowledge pertaining to zoonotic diseases (Table-4). The result specifies that dog owners with more or less housing space had with homogeneous knowledge level about zoonotic diseases. Thus, null hypothesis (Ho_1_) was accepted in case of housing space and it was concluded that housing space exerted non-significant influence in determination of knowledge about zoonotic diseases [5,8,9].

### Extension contact and knowledge

The extension contact of dog owners had positive and significant relationship (r=0.522**) with their knowledge about zoonotic diseases, which implies that extension contact of dog owners play vital role in determining their knowledge about zoonotic diseases (Table-4). The knowledge about zoonotic diseases was observed higher among those dog keeper having a higher level of extension contact and vice-versa. Thus, null hypothesis (Ho_1_) was rejected in case of extension contact and concluded that there was positive and significant relationship between extension contact of dog owners and their knowledge about zoonotic diseases [5,10-12]. Reports suggest that veterinarians play an important role and serve as a source of extension contact to provide knowledge to dog-owners regarding health and management aspects [13].

### Mass media exposure and knowledge

The mass media exposure of dog owners had positive and significant relationship (r=0.205*) with their knowledge of zoonotic diseases (Table-4). The result reflects that mass media played an important role in improving knowledge of the dog owners regarding zoonotic diseases. More coverage related to the special zoonotic diseases related programs in different media, tendency of the owners to use mass media more for entertainment and also because of the college level education played role for using of the mass media in urban areas. Thus, null hypothesis (Ho_1_) was rejected in case of mass media exposure and concluded that there was the significant influence of mass media exposure of dog owners in shaping knowledge about zoonotic diseases [11,12,14].

### Management orientation and knowledge

The management orientation of dog owners had positive and significant relationship (r=0.264**) with their knowledge about zoonotic diseases (Table-4). The result indicates that the knowledge about zoonotic diseases was observed better among those dog owners who had a high degree of management orientation as compared to those who had a low degree of management orientation. The results indicate that owners with the better ability to plan the activity to get best from the available resources leads to have better knowledge about zoonotic diseases. It is natural that person with good management ability will always try to collect useful information and convert them into knowledge as a useful input. Thus, null hypothesis (Ho_1_) was rejected in case of management orientation and concluded that there was positive and significant influence of management orientation of dog owners on their knowledge about zoonotic diseases.

## Conclusion

Independent variables, *viz*., education, experience in dog keeping and housing space were observed to have negative and non-significant relationship with knowledge of dog owners about zoonotic diseases. The three independent variables, *viz*., extension contact, mass-media exposure and management orientation had a significant relationship with knowledge of dog owners about the zoonotic importance of canine scabies and dermatophytosis. Data provides new insights which would aid in awareness programs on zoonotic disease prevention in urban areas of Gujarat state.

## Authors’ Contributions

HSR: Prepared, revised and drafted the manuscript which is a part of the M. V. Sc. research work and includes experimental design, collection of data by personal interview and statistical analysis; JBN & BMP: Provided guidance throughout the study period as well as helped in academic/legislative aspects; CMB: Provided contacts of dog owners in Anand area and assisted in the survey work.
